# Joining Properties of SPFC440/AA5052 Multi-Material Self-Piercing Riveting Joints

**DOI:** 10.3390/ma15092962

**Published:** 2022-04-19

**Authors:** Ze-Jie Zhou, Zhi-Chao Huang, Yu-Qiang Jiang, Nan-Lin Tang

**Affiliations:** Key Laboratory of Conveyance and Equipment, Ministry of Education, East China Jiaotong University, Nanchang 330013, China; cancyzhou@163.com (Z.-J.Z.); jiangyq2021@126.com (Y.-Q.J.); tang_nanlin@163.com (N.-L.T.)

**Keywords:** aluminum alloy, high-strength steel, self-piercing riveting, static strength, fatigue strength, failure mode

## Abstract

With the development of new energy vehicles, the joining of lightweight alloys has received more attention. Self-piercing riveting experiments of aluminum alloy and high-strength steel sheets were performed to analyze the effects of rivet height and laying order of metal sheets on the joining quality in the work. The forming surface, cross-sectional morphology, static tensile property, fatigue property, failure mode, and mechanism were analyzed. The results show that AA5052 alloy and SPFC440 steel can be joined effectively by self-piercing riveting, and there is good contact between rivet head and sheet surfaces. When the rivet is 2.5–3.5 mm higher than the total thickness of two layers sheets, the rivet leg flares symmetrically without cracks or buckling, and the lower sheet completely encapsulates the joint button. The joints have better static tensile properties when the rivet is about 3 mm higher than the thickness of two sheets. The higher static strength is obtained when the aluminum alloy is placed at the lower position. The rivet legs fall off from the lower sheets for all the samples in the tensile tests, which is independent of the rivet height and laying order of metal sheets. The fatigue strength of the sample with the rivet height of 7 mm is the greatest, and the fatigue cracks always occur on the aluminum sheet under all experimental conditions. The findings in this work can help the practical application of self-piercing riveting for aluminum/steel sheets.

## 1. Introduction

The light-weighting of the automobile body means the weight reduction is as much as possible under the premise of strength and safety performance [1,2]. The use of advanced materials is the most important means to reduce body weight [3]. These advanced materials can be divided into two main categories. One is high-strength steel. Using high-strength steel sheets can not only reduce the thickness of the structure, but also ensure the performance of automobiles, thus reducing the weight of the car [4,5,6]. The other is low-density materials, such as Al alloy [7,8,9], Mg alloy [10,11], Ti alloy [12,13], and composites [14,15,16]. These similar or dissimilar materials will inevitably face the joining challenges in practical applications. It is difficult to weld high-strength steel and aluminum alloys due to their great differences in physical and mechanical properties. The formation of hard and brittle metal compounds in the welding process can also reduce the mechanical properties of the joints [2,17]. It is urgent to develop new methods to join these materials.

In the self-piercing riveting (SPR) processing, the rivet is driven by the SPR machine and penetrated in the upper sheet and then spared in the lower sheet, and an effective mechanical self-locking is formed. The SPR processing can realize the connection of two or more layers of sheet metal. The rivet penetrates the upper sheet metal under the action of the punch and spares in the lower sheet. The SPR joint has the effect of mechanical self-locking. SPR has the advantages of no spark, low energy consumption, no pre-drilled holes, no heat effect, short riveting procedure, low noise, and the ability to join multi-layer or mixed materials [9,18]. The conventional welding methods are gradually replaced by new SPR processing [19]. Bang et al. [17] joined A356-T6 aluminum alloy and SAPH440 automotive steel sheets using friction stir spot welding and SPR, and found that the tensile-shear strength of the SPR joints is higher than that of the weld joints. Deng et al. [20] reported the effect of die geometry on the mechanical response of the SPR joints, and the results indicated that good surface quality can be obtained when the conical section dies with a moderate convex were used during the SPR process. Zhao et al. [21] studied the low-velocity impact behaviors of SPR joints, and found that the low-velocity impact can reduce the fatigue lives of the SPR joints. Jia et al. [5] studied the fatigue life of the DP590-AA6061 sheets SPR joints, and their results proved that the fatigue life of the SPR joints was improved by reducing the micro-vibration wear. Du et al. [22] investigated the SPR join ability of B280VK and 6063-T6 sheets, and found the SPR joinability of 6063/B280VK can be improved by setting the softer and thicker sheet as the lower sheet when the flow stress of upper sheet is equal from 183 MPa to 517 MPa. Abe et al. [23] joined JSC780 and AA5052 sheets using the SPR process, and the corrosion behavior and the joint strength were measured by salt spray tests. The results indicated that the thickness reduction occurred near the minimum thickness of the upper sheet and in the upper surface on the edge of the lower aluminum alloy sheet, whereas the top surface of the upper sheet and the upper surface of the lower sheet were mainly corroded in the riveted joint. Deng et al. [24] developed a thermally-assisted SPR process to improve the properties of AA6061-T6 and DP980 alloy joints, and found that appropriate heating can produce crack-free joints. Zhang et al. [25] investigated the static tensile and fatigue strength of an aluminum–steel SPR joint, and revealed that the mechanical and fatigue performances of the SPR joints were improved when the thickness of the steel sheet was increased. Du et al. [26] joined AA6111 and DP780 by the SPR process, and the effects of fatigue load on energy absorption and remaining static strength properties were studied. The results indicated that the energy absorption capacity of the specimens decreased significantly after high cycle fatigue, and fretting wear was found at the contact area between the rivet and aluminum sheets. Han et al. [27] examined the effect of coatings on the quality of NG5754 and AA5182 SPR joints, and found that the presence of coating can affect the joint quality in terms of head height, interlock distance, and remaining material thickness. Ma et al. [28] investigated the effects of the rivet and die combinations on the rivetability and mechanical properties of mild steel CR4 and AA6061-T6 joints. The results indicated that the softer rivets and larger dies can improve the rivetability range, but decrease the joint strength. The longer rivets and smaller dies can narrow down the rivetability range and increase the joint strength. Abe et al. [4] joined one thin 5000 series aluminum alloy sheet and two thin 980 MPa ultra-high-strength steel sheets by SPR processes. The results indicated that three sheets can be successfully joined by optimizing the shapes of the die and rivet. Achira et al. [6] joined three thin sheets of 980 MPa steel and AA5052 alloy, and the effect of the laying order of the three sheets on the joining quality was studied. The results indicated that the joining range was relatively wide when the lower sheet was A5052, and the rivet leg spread out moderately without defects when the upper and lower sheets were aluminum alloy, and the middle sheet was steel.

Due to their excellent forming ability, corrosion resistance, and strength, AA5052 aluminum alloy and SPFC440 high-strength steel are widely used in automobile bodies. However, there are few studies on SPR joints of AA5052 and SPFC440 mix-materials. How to optimize the rivet heights and laying order of metal sheets is essential for cost reduction and joint strength. The self-piercing riveted AA5052 aluminum alloy and SPFC440 high-strength steel were studied, and the effects of process parameters on the forming property, static tensile, and fatigue properties of the SPR joint were studied in the work. The research results are expected to expand the application and popularization of aluminum/high-strength steel metals in automobile bodies, especially in the field of new energy vehicles.

## 2. Materials and Methods

### 2.1. Materials

The alloy used in the work are 150 mm × 36 mm × 2.5 mm AA5052 aluminum alloy (Shanghai Juli Metal Products Co., Ltd., Shanghai, China) and 150 mm × 36 mm × 2 mm SPFC440 high-strength steel (Dongguan Zhenghai Mould Steel Co., Ltd., Dongguan, China). The chemical composition and mechanical properties of AA5052 alloy and SPFC 440 steel are shown in Table 1 and Table 2, respectively, which were obtained from the manufacturer.

### 2.2. Methods

**Figure 1:**
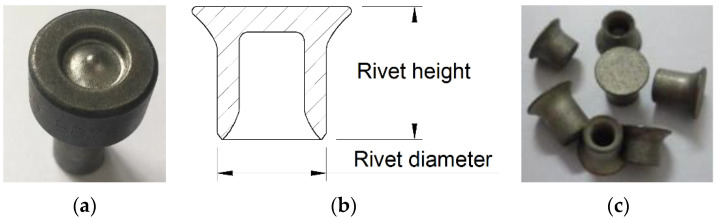
The die and rivet used in this work: (**a**) Boss-concave die; (**b**) Rivet geometry; (**c**) Semi-tubular rivet.

The RV300023 hand-held SPR machine (Henrob Limited, Deeside, UK) was employed to get SPR joints. The boss-concave die and Φ 5.3 mm semi-tubular rivets were used in the test, as shown in Figure 1. The SPR tests were carried out with rivet heights of 6 mm, 7 mm, 8 mm, and 9 mm to evaluate the influence of rivet heights on the joining quality of aluminum/steel dissimilar materials.

**Figure 2:**
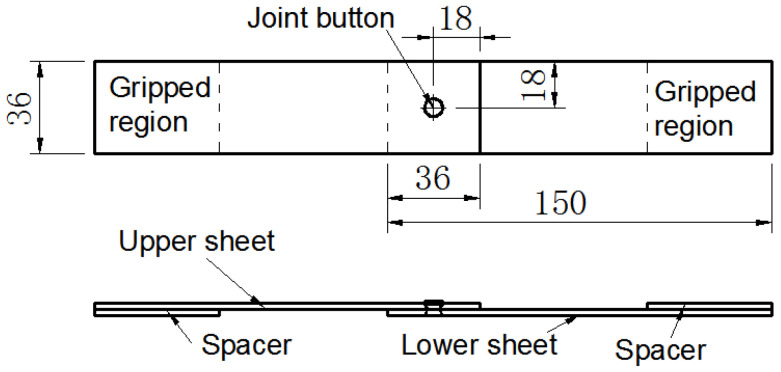
The size of the lap-shear SPR joint (Unit: mm).

Auxiliary heating of sheet metal before riveting can improve the plasticity of metal and the quality of connection [8]. Cars are usually mass-produced; increasing the number of processes will increase the production cycle and cost. Considering the strength level of AA5052 and SPFC440, the joining of aluminum/steel is carried out at room temperature. The SPR joint sizes are displayed in Figure 2. The scheme proposed in Figure 2 creates the torsion stress in the joint region. Therefore, the spacers with a corresponding thickness were used to minimize the torsion stress on the gripped region. All specimen designs and joint tests referred to GB/T 228.1-2010 (Metallic Materials Tensile Test—Part 1: Room Temperature Test Method), GB 50018-2002 (Technical Code of Cold-formed Thin-wall Steel Structures), and GB 2649 (Methods of Sampling for Mechanical Properties Tests of Welded Joint). At the same time, the designs and tests also referred to the research of other scholars [29]. To reveal the effects of laying order of metal sheets on properties of SPR joints, two types of laying order of metal sheets were adopted: the upper sheet is SPFC440 and the lower sheet is AA5052 (i.e., SPFC-AA joints); the upper sheet is AA5052 and the lower sheet is SPFC440 (i.e., AA-SPFC joints).

The effects of the riveting process on the quality of SPR joints are analyzed from surface morphology, the cross-sectional profiles, static tensile strength, fatigue strength, and failure mode of the SPR joints. These aspects are the key research interests of the experts and scholars who are engaged in SPR-related works [30,31]. The cross-section of SPR joints was observed and measured by a Dino-Lite-AM3111T versatile digital microscope (Weidi Optical (Wuxi) Co., Ltd., Wuxi, China). The static tensile strength was tested by the RGM4030 universal testing machine (Shenzhen Regal Instrument Co., Ltd., Shenzhen, China). The fatigue testing machine (QBG-50, Changchun Qianbang Testing Equipment Co., Ltd., Changchun, China) was used for the fatigue tests. The loading form in the fatigue test was tension/tension loading, the stress ratio was 0.1, and the frequency was 88 Hz. The fatigue fracture surfaces were observed by an SU8010 scanning electron microscope (Hitachi High-Technologies Co., Ltd., Tokyo, Japan). The chemical element of the fretting surface was analyzed using an X-Flash 6160 energy dispersive spectrometer (Bruker Co., Ltd., Bremen, Germany).

## 3. Results and Discussion

The quality of the SPR joints directly affects the application of the SPR technology in practical productions. The forming quality of the SPR joint is affected by rivet height, the geometry of the die, and the material properties of sheets. The joining quality can be evaluated from the surface morphology, central cross-section profile, static tensile properties, fatigue properties, fracture conditions, and so on [32].

### 3.1. Surface Morphology of SPR Joints

**Figure 3:**
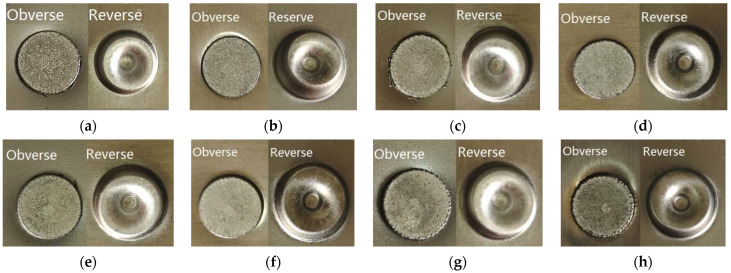
Surfaces of SPR joints: (**a**) SPFC-AA joint with 6 mm rivet; (**b**) AA-SPFC joint with 6 mm rivet; (**c**) SPFC-AA joint with 7 mm rivet; (**d**) AA-SPFC joint with 7 mm rivet; (**e**) SPFC-AA joint with 8 mm rivet; (**f**) AA-SPFC joint with 8 mm rivet; (**g**) SPFC-AA joint with 9 mm rivet; (**h**) AA-SPFC joint with 9 mm rivet.

Figure 3 shows the morphologies of the obverse and reverse of the SPR joint buttons. Regardless of the rivet height and the laying order of sheets, both the obverse and reverse of the joint buttons are formed well, and no obvious cracks or other defects are found. Good contacts can be seen between the rivet head and the surface of the sheet.

### 3.2. Central Cross-Sectional Profiles Characteristics of SPR Joints

**Figure 4:**
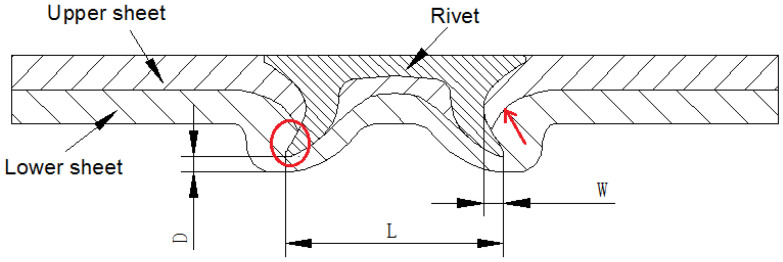
Cross-sectional profiles of an SPR joint.

The central cross-sectional profiles of the SPR joints have important influences on the quality of the SPR joints [10,18,33]. The key parameters of a central cross-sectional profile are defined in Figure 4. W is the length of the internal lock. L is the expanding degree of the rivet leg. D is the residual thickness of the bottom. The rivet leg should be flared symmetrically without cracks or buckling, so the force on the rivet is evenly distributed during service. The length of the internal lock should be large enough to ensure the mechanical self-locking effect of the SPR joints. A certain amount of residual thickness of the bottom should be retained to prevent stress concentration and surface defects.

**Figure 5:**
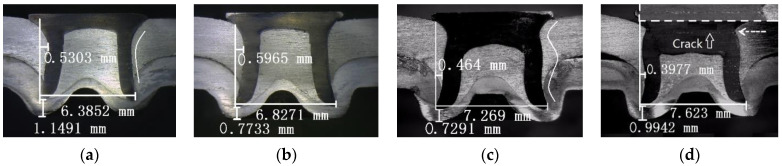
Cross-sectional profiles of AA-SPFC joints at rivet heights of (**a**) 6 mm; (**b**) 7 mm; (**c**) 8 mm; (**d**) 9 mm.

**Figure 6:**
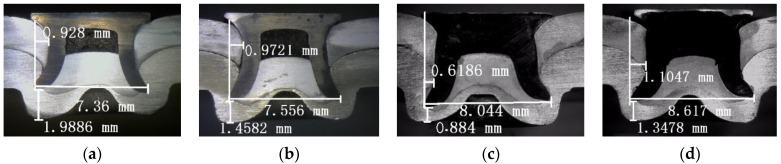
Cross-sectional profiles of SPFC-AA joints at rivet heights of (**a**) 6 mm; (**b**) 7 mm; (**c**) 8 mm; (**d**) 9 mm.

Figure 5 and Figure 6 are the cross-sectional profiles of the SPR joints with different rivet heights. It can be observed that the three indexes (W, L, and D) of SPFC-AA samples are all larger than that of AA-SPFC samples at different rivet heights. These phenomena are ascribed to the softer aluminum alloy lower sheets. The rivet leg meets less deformation resistance when penetrating the AA5052 sheet. When the harder SPFC440 sheet is placed in the lower sheet, the expansion resistance of the rivet leg is increased when penetrating the high-strength steel, and it is difficult for rivets to achieve full flare. Therefore, the expanding degree of the rivet leg and the length of the internal lock of SPFC-AA joints are bigger. Due to the full expansion of the rivet legs, the residual thickness of the bottom increases accordingly.

It can be also observed that the expanding degree of rivet leg increases gradually with the increase of rivet height, regardless of the laying order of sheets. However, the other two indexes (W and D) have no obvious regularity, which is related to the inconsistency of rivet head thickness. It can be observed from Figure 5a,b and Figure 6a,b that the rivet leg smoothly expands outward into the lower sheet, and a “C” shape of the rivet leg is formed, as shown by the line in Figure 5a. In this case, the rivet head and the outer wall of the rivet leg transits smoothly, and there is no greater stress concentration. Figure 5c,d and Figure 6c,d show that the rivet leg undergoes upsetting and wave-shaped buckling, as marked by the line in Figure 5c. The buckling transition of the wave shape is smooth when the rivet height is 8 mm. However, a sharp angle less than 90 degrees is formed between the rivet head and the outer wall of the rivet leg for the 9 mm rivet, as indicated by the dotted arrow in Figure 5d. When the rivet height is 9 mm, there is an obvious crack in the rivet. The enlarged crack is shown in the upper right corner of Figure 5d. The direction of the crack is perpendicular to the direction of riveting force, that is, the direction of rivet motion, and is located at the junction of rivet head and rivet leg. This is because the sudden change of the rivet sections at this point brings about greater stress concentration. The rivet is subject to great resistance in the process of penetrating the steel sheet, and the cracks are easily formed. As can be seen from Figure 5d and Figure 6d, there is a large gap between the rivet head and the surface of the upper sheet.

The SPR process with semi-tubular rivets can realize the connection of the aluminum alloy and the high-strength steel sheet. More attention should be paid to the relationship between the rivet height and the total thickness of metal sheets. The total thickness of the aluminum alloy and steel sheet is 4.5 mm in the work, the joint morphologies are good, and no obvious defects are found when the rivet height is 6~8 mm. It can be concluded that when the rivet height is about 1.5~3.5 mm higher than the total thickness of sheet metals, an excellent SPR forming quality can be acquired. However, when the rivet height is more than 4.5 mm higher than the total thickness of sheet metals, the joint has obvious forming defects, such as non-smooth deformation and cracks.

### 3.3. Results and Analysis of Static Tensile Tests

Static tensile strength is an important mechanical property of SPR joints. Static tensile tests with different rivet heights and laying orders of metal sheets are performed in the work, and their influence on joining quality is analyzed.

#### 3.3.1. Static Tensile Strength of SPR Joints

**Figure 7:**
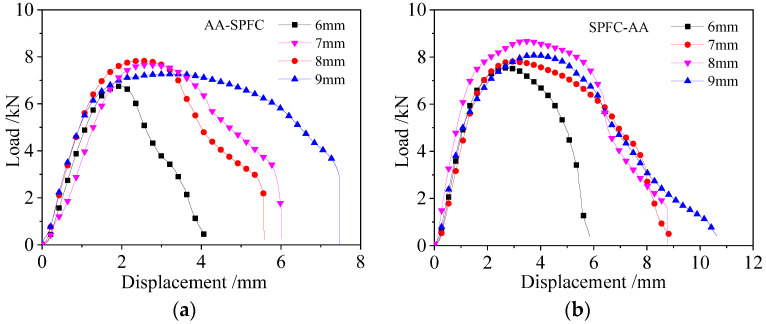
Displacement–load curves of SPR joints: (**a**) AA-SPFC joints; (**b**) SPFC-AA joints.

Figure 7 shows the static tension displacement–load curves of SPR joints with different rivet heights, and Figure 7a,b are riveted joints with AA-SPFC and SPFC-AA alloys, respectively. As can be seen from Figure 7, the load increases with the displacement, and then decreases until a peak load is reached. The peak load is used as the evaluation index of the static tensile properties of riveted joints in this work. The peak load in the displacement–load curve is also used to evaluate the static tensile properties of self-piercing riveting joints by other researchers [14,34,35]. 

The static tensile curve can be divided into three stages. In the initial stage of the tensile test, the tensile load increases linearly with the increase of displacement. The SPR structure undergoes elastic deformation at this stage. The slopes of the displacement–load curves hardly change with the rivet height when the laying order of metal sheets is fixed. The slopes of SPFC-AA specimens are higher than that of the AA-SPFC specimens. As discussed earlier, the length of the internal lock and the expanding degree of the rivet leg of the SPFC-AA joints are larger than those of AA-SPFC joints, so the SPFC-AA joint has stronger self-locking ability and a tighter joint. It is also shown in reference [15] that the displacement–load curve has a higher slope because of its tighter structure. The slope of the displacement–load curve with the 8 mm rivet is slightly higher than that of the other curves for the SPFC-AA specimens, which is due to the tighter contact between the rivet and the metal sheets (Figure 6c). The metal does not fill the rivet cavity when the rivet height is 6 mm (Figure 6a) and 7 mm (Figure 6b), which results in a large gap in the rivet inner cavity. There is a big gap between the rivet head and the upper sheet when the rivet height is 9 mm (Figure 6d).

In the second stage, the load increases slowly and non-linearly with the increase of displacement, and then gradually reaches the maximum load value. The specimen undergoes elastic–plastic deformation, and the upper sheet undergoes warping deformation. When the rivet heights are 6 mm, 7 mm, 8 mm, and 9 mm, the maximum load values of AA-SPFC joints are 6.75 kN, 7.67 kN, 7.83 kN, and 7.27 kN, and the corresponding displacements are 1.92 mm, 2.67 mm, 2.52 mm, and 2.81 mm, respectively. The maximum load values of SPFC-AA joints are 7.55 kN, 7.82 kN, 8.68 kN, and 8.08 kN, and the corresponding displacements are 2.78 mm, 2.80 mm, 3.36 mm, and 3.70 mm, respectively. The maximum loads are higher when the rivet heights are 7 mm and 8 mm, regardless of the laying order of the sheets, but lower when the rivet height is 6 mm. This is related to the self-locking ability of rivet in sheet metal. When the rivet heights are 7 mm and 8 mm, the length of the internal lock is larger than that of 6 mm, so the locking effect and the static tensile performance are perfect. When the rivet height is 9 mm, the deformation of the rivet is serious, and a crack appears (Figure 5d), causing the dropped peak load. The maximum load of SPFC-AA joints is higher than that of AA-SPFC joints under the same SPR forming parameters. The softer AA5052 aluminum alloy serves as the lower sheet for the SPFC-AA joint, and the rivet encounters smaller deformation resistance when penetrating the lower sheet. As such, the flared deformation of the rivet in the lower sheet is greater, and the self-locking effect of the joint is better, leading to the high tensile property.

The third stage is the plastic deformation and damage stage. The load decreases rapidly and non-linearly with the increase of displacement. The upper sheet warps and deforms more seriously, and the rivet inclines gradually in the sheets. The flared rivet tail shrinks gradually under the action of tensile force. The displacement of the SPR structure is the smallest when the rivet height is 6 mm before the sample is completely destroyed. This is because the length of the internal lock and the expanding degree of the rivet leg are minimal, and self-locking is poor. The difference in the decline trends of displacement–load curves is not significant when the rivet heights are 7 mm and 8 mm. The displacement–load curves of AA-SPFC joints with 9 mm have a significant difference. The main reason is that the tested curve has a large displacement in the elastic–plastic stage. This may be due to the deformation of the rivet. The rivet tail is seriously deformed in the SPR process, whereas the deformed rivet is recovered partly, and some energy is released in the static tensile tests. The displacement of SPFC-AA joint is larger than that of AA-SPFC joint when the rivet height is the same, which indicates that SPFC-AA joints can absorb more energy and have good self-locking effects. At last, the rivet leg is pulled out from the lower sheet. The load drops rapidly, and the self-locking structure is completely destroyed. Not all specimens have the distinct pull-out phenomena, and only AA-SPFC joints with rivet heights of 7 mm, 8 mm, and 9 mm have distinct pull-out failure stages.

#### 3.3.2. Failure Forms of SPR Joints

**Figure 8:**
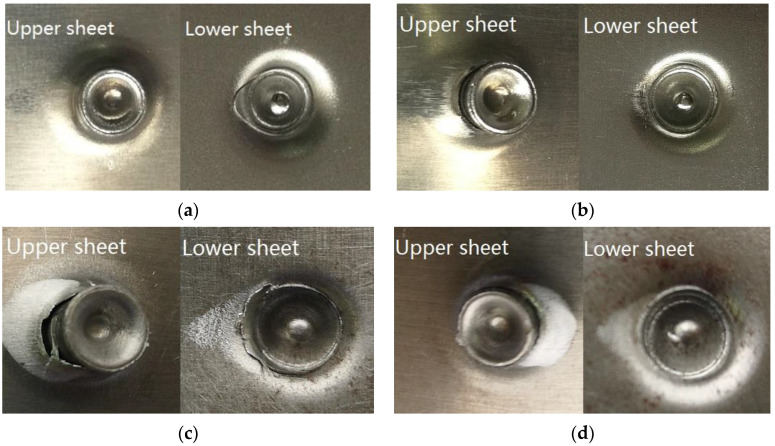
Failure forms of AA-SPFC joints at rivet heights of (**a**) 6 mm; (**b**) 7 mm; (**c**) 8 mm; (**d**) 9 mm.

**Figure 9:**
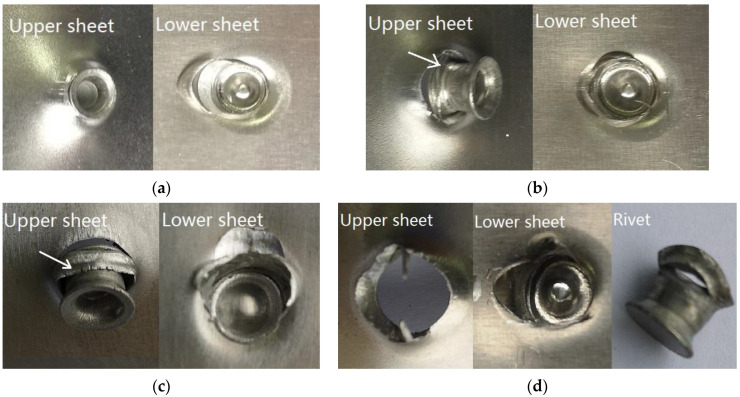
Failure forms of SPFC-AA joints at rivet heights of (**a**) 6 mm; (**b**) 7 mm; (**c**) 8 mm; (**d**) 9 mm.

Figure 8 and Figure 9 show the failure forms of the SPR joints with different rivet heights. It can be seen from Figure 8a–d and Figure 9a–c that the failure forms of SPR joints after static tension are the pulling out of the rivet leg from the lower sheet. For Figure 9d, the rivet is pulled out from both the upper sheet and the lower sheet. In the SPR process, the rivet head is stuck outside the upper metal sheet, and the rivet leg is pierced into the lower metal sheet. Despite the plastic deformation of the rivet leg and its flare into a trumpet shape, the joining force between the rivet head and the upper sheet is greater than the joining force between the rivet leg and the lower sheet. Since the rivet head fastens the upper sheet, the rivet leg does not eventually pierce through the lower sheet, and forms a reverse lock on the back surface of the lower sheet. At the same time, the deformation resistance of the rivet head is also greater than that of the rivet leg, so the rivet leg is easily pulled off from the lower sheet.

The self-locking phenomena of SPR joints are more pronounced when the aluminum alloy sheet acts as the lower sheet. As such, the force that pulls out of the rivet leg from the aluminum sheet is greater, and the rivet cuts off the upper sheet at the location below the rivet head, as marked by the arrow in Figure 9b,c. The rivet falls off from the upper sheet at last, as shown in Figure 9d. The self-locking is slightly worse when the rivet height is small, so there is no phenomenon in which the rivet head cuts off the upper sheet, as shown in Figure 9a. It can also be concluded that the static tension failure of the SPR joints in this case mainly depends on the self-locking of the rivet leg in the lower sheet.

### 3.4. Fatigue Performance of SPR Joints

Fatigue tests are performed to compare the fatigue performance of SPR joints. In this work, a tension–tension loading mode is adopted, the maximum load is 5 kN, and the stress ratio is R = 0.1. Fatigue tests are performed on the SPR joints with different rivet heights and laying orders of sheets.

#### 3.4.1. Fatigue Strength

Six samples are tested at each test parameter. Each sample is loaded until the occurrence of fatigue failure, and six fatigue lives are obtained. The fatigue performance is analyzed by calculating the average value of six fatigue lives. The fatigue test results are shown in Table 3.

The average fatigue lives of different samples can be seen in Table 3. Regardless of the laying order of the sheets, the fatigue life increases first, and then decreases with the increase of rivet height. When the rivet height is 7 mm, the fatigue life reaches its peak value. The fatigue life of the AA-SPFC sample is 209.6 × 10^3^ cycles, whereas the fatigue life of the SPFC-AA sample is 111.3 × 10^3^ cycles. When the rivet height is 9 mm, the fatigue life reaches its lowest value. The fatigue life of the AA-SPFC sample is 45.2 × 10^3^ cycles, whereas the SPFC-AA sample is 56.4 × 10^3^ cycles. When the rivet height is 7 mm, the rivet height is about 2.5 mm higher than the total thickness of sheet metals. In this case, the self-locking is stronger than that of the rivet height of 6 mm. Therefore, the fatigue strength of the SPR joints with 7 mm rivet height is higher than that of 6 mm rivet height. When the rivet height is further increased, the rivet leg cannot spare smoothly in the metal sheet, and the upsetting and wave-shaped buckling deformation appears. The higher the rivet is, the more obvious the uneven deformation is, as displayed in Figure 5 and Figure 6. As a result, when the rivet height increases from 7 mm to 9 mm, the pressure between sheets and the rivet, and between the upper sheet and lower sheet, increases. When the contact stress increases, the friction and wear increase, leading to the increased probability of crack initiation and propagation, and decreased fatigue life.

When the rivet heights are 6 mm and 9 mm, the laying order of metal sheets has no significant effect on the fatigue life, as shown in Table 3. However, when the rivet heights are 7 mm and 8 mm, the fatigue life of the AA-SPFC samples is higher about 50% than that of SPFC-AA samples. In general, the laying order of the sheets and the material properties determine the initial position of fatigue cracks in the joint. In the SPR process, the rivet and part of the upper sheet are forced into the lower sheet, and serious tensile plastic deformation and large residual tensile stress occur in the lower sheet. Therefore, the initiation and propagation of cracks on the lower sheet will be intensified under fatigue loading. When the aluminum alloy serves as a lower sheet, the wear resistance of the aluminum sheet is worse than that of high-strength steel. Under the combined action of friction, wear, and tension stress, fatigue failure occurs quickly, and the fatigue life is decreased. Even if the lower sheet is also affected by friction, wear, and tensile stress in the fatigue tests, the fatigue cracks do not occur easily when the lower sheet is high-strength steel, which has good wear resistance. The expanding degree of the rivet leg of the SPFC-AA joint is larger than that of the AA-SPFC joint, as shown in Figure 5 and Figure 6. This indicates that the self-locking force of the SPFC-AA joint is larger, which leads to the increased pressure and contact stress between sheets and the rivet, so the fatigue life of the SPFC-AA joint is lower than that of the AA-SPFC joint.

#### 3.4.2. Fatigue Failure Mechanism of AA-SPFC Joints

**Figure 10:**
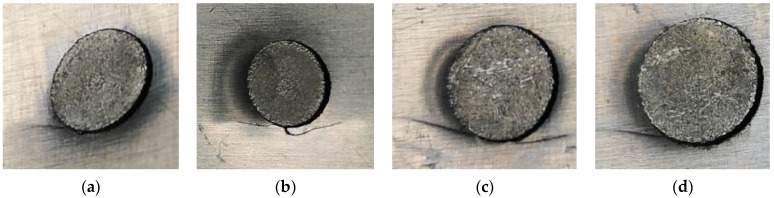
Failure forms of AA-SPFC joints with rivet heights of (**a**) 6 mm; (**b**) 7 mm; (**c**) 8 mm; (**d**) 9 mm.

The surface morphologies of fatigue failure for AA-SPFC are shown in Figure 10. The fatigue failure occurs in the aluminum alloy sheet, and the cracks are located near the rivet head, as shown in Figure 10. The direction of the crack is approximately perpendicular to the direction of the fatigue load.

**Figure 11:**
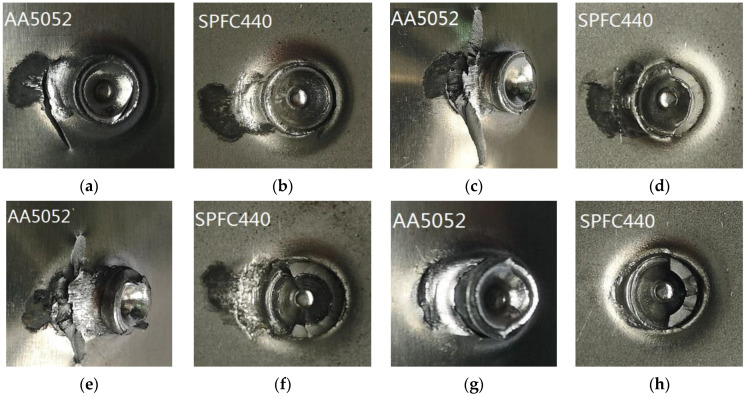
Fretting morphologies of AA-SPFC joints: (**a**) AA5052 with 6 mm rivet; (**b**) SPFC with 6 mm rivet; (**c**) AA5052 with 7 mm rivet; (**d**) SPFC with 7 mm rivet; (**e**) AA5052 with 8 mm rivet; (**f**) SPFC with 8 mm rivet; (**g**) AA5052 with 9 mm rivet; (**h**) SPFC with 9 mm rivet.

In the fatigue test, when the load is decreased by 10% of the initial given load or the frequency is reduced by 6 Hz, the fatigue tests stop immediately. Therefore, the samples will not break apart completely, and the fatigue cracks of some samples are not extended to the outer surface of the sheets. This is the case for AA-SPFC samples. Some fatigue cracks on the outer surface of the upper sheet cannot be seen clearly. A clear fatigue fracture can be observed from Figure 11 when the sample is broken.

Figure 11 shows the cracks of AA-SPFC samples after fatigue load. Fretting wear of aluminum and steel sheets occur under fatigue load. The abrasion resistance, hardness, strength, and fatigue properties of high-strength steel are significantly better than those of aluminum alloy. Therefore, fatigue cracks are easily formed in the AA5052 sheet under the combined action of stress and fretting. There are obvious fretting marks on the contact surface of aluminum sheets (Figure 11a,c,e,g) and steel sheets (Figure 11b,d,f,h), and the fatigue cracks on the aluminum sheet originate from the fretting area. When the contact body of steel and aluminum sheets is subjected to alternating fatigue load, the alternating fatigue load will cause the contact interface to move slightly. The fresh metal surface is exposed at the position of the contact surface. The metal surface is affected by oxygen and water in the air, and oxide films are formed. When the oxide film ruptures, an abrasive debris is formed. Abrasive debris accumulates and peels off in the fatigue tests, and the abrasive pit is formed. The abrasive pit is located at the tensile stress position, so the fatigue crack originates in the abrasive pits.

The friction degree of SPR samples with 7 mm (Figure 11c,d) and 8 mm (Figure 11e,f) rivets are more serious than that of 6 mm (Figure 11a,b) and 9 mm (Figure 11g,h) rivets. From Table 3, the fatigue life of SPR samples with 7 mm and 8 mm rivet heights is longer, so the number of fatigue cycles is larger, and the friction degree is more serious, whereas the friction degree of SPR samples with a rivet height of 6 mm is less. The fatigue life is the shortest and the friction degree is the lightest when the rivet height is 9 mm. Moraes et al. [36] revealed that the failure of SPR joints occurs at locations away from the rivet. The location of the crack in the work of Moraes is not consistent with this work, which is related to a different riveted material. However, the fatigue failure mechanism in the work of Moraes is consistent with this work. Fretting wear occurs at the location of the fatigue crack initiation.

**Figure 12:**
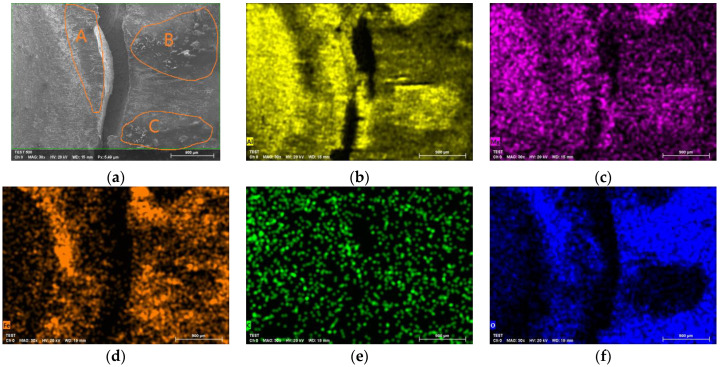
Energy permissive analysis of fatigue fracture in AA5052 sheet: (**a**) Fatigue crack in lower surface of AA5052 sheet (A, B, C are typical serious friction, oxidation, and corrosion areas); (**b**) Distribution of chemical element aluminum; (**c**) Distribution of chemical element magnesium; (**d**) Distribution of chemical element ferrum; (**e**) Distribution of chemical element carbon; (**f**) Distribution of chemical element oxygen.

The AA-SPFC specimen with the 6 mm rivet is selected for the chemical element analysis, as can be seen in Figure 12. Figure 12a shows the fatigue crack on the lower surface of the AA5052 sheet. Figure 12b–f shows the chemical element distribution near the fatigue crack. The chemical elements include aluminum, magnesium, ferrum, carbon, oxygen, and so on. As can be seen from the map of the oxygen element, there are serious friction, oxidation, and corrosion in areas A, B, and C. AA5052 is a kind of Al-Mg alloy, so there are elements of aluminum and magnesium on the wear surface. The steel sheet and the aluminum alloy sheet are scraped against each other in the fatigue test, so the chemical element ferrum can be seen on the aluminum sheet. The carbon content of SPFC440 is about 0.24%, which is very low, and the weight of carbon is very small. It is difficult to measure carbon accurately using energy dispersive spectrometer (EDS) equipment. There are two possible reasons for the presence of carbon. Firstly, the sample is contaminated slightly during the preparation process. Secondly, it is affected by the sensitivity of EDS equipment. The presence of oxygen may be related to the following factors. Firstly, oxygen comes from initial materials. The chemical property of aluminum is very reactive, and readily oxidizes in air. AA5052 is oxidized and covered with a dense Al_2_O_3_ oxide film layer. FeO, Fe_2_O_3,_ or Fe_3_O_4_ are also present on the surface of SPFC440 metal. Secondly, the existence of oxygen is related to the fatigue tests. Aluminum alloy and the steel sheet are scraped against each other in fatigue tests, so the oxide films on the metal surface are worn away to reveal the pure metal inside. The pure metal is further oxidized in the air, producing a new oxide film, which is then worn away again. The oxide film on the metal surface experiences wearing and producing, which leads to the appearance of oxygen on the worn surface. Thirdly, the existence of oxygen is related to the preparation, preservation, and transportation of the sample. If the sample is not properly stored at these points, oxygen corrosion may occur. The presence of oxygen may also be associated with EDS equipment. Limited by the sensitivity of the EDS, it can also lead to the presence of oxygen on the energy spectrum maps. Steel and aluminum surfaces contact each other directly in the SPR joint. There is a large electrode potential difference between aluminum and steel, and the aluminum–steel contact pair is prone to electrochemical corrosion. The dense oxide film layer on the surface of aluminum alloy can prevent electrochemical corrosion to some extent. However, in the fatigue test, the oxide film on the surface is worn and destroyed, and the pure metal is exposed. AA5052 aluminum alloy with lower electrode potential is more prone to anodic oxidation. 

**Figure 13:**
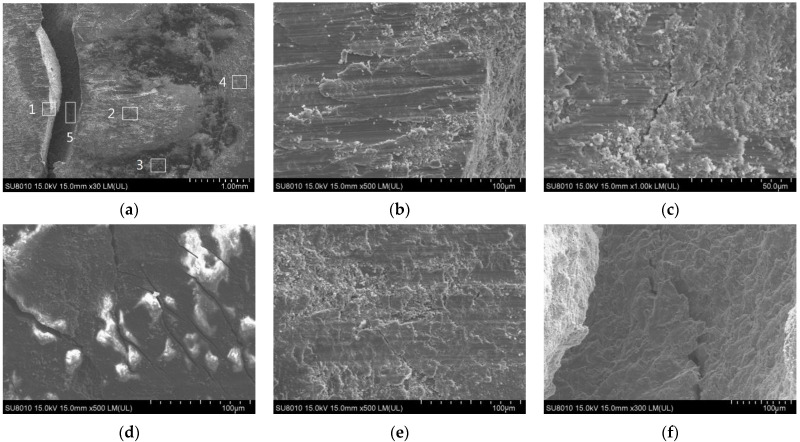
Fatigue wear surface of AA5052 sheet: (**a**) Morphology of fatigue fracture (The locations marked by 1,2,3,4,5 are enlarged in subfigure (**b**–**f**)); (**b**) Fracture morphology of zone 1; (**c**) Fracture morphology of zone 2; (**d**) Fracture morphology of zone 3; (**e**) Fracture morphology of zone 4; (**f**) Fracture morphology of zone 5.

The fatigue wear surface of the aluminum sheet in AA-SPFC samples is taken as an example to analyze the fretting behavior. To study the mechanism of fatigue fracture failure deeply, the fretting regions of 1, 2, 3, 4, and 5 in Figure 13a are amplified, respectively. Zone 1 (Figure 13b) is the edge of the fatigue crack. There are obvious large flakes of abrasive debris and some fine particles of metal oxide debris. The ploughing phenomena can be seen in zone 2 (Figure 13c), and obvious fatigue micro-cracks can also be observed. Zone 1 and zone 2 are located in the abrasive pit. The characteristics of significant oxidation are shown in Zone 3 (Figure 13d), and obvious cracks can be seen. In zone 4 (Figure 13e), oxide layers are further crushed into fine layers and debris. At the same time, there are a large number of micro secondary cracks in the main fatigue crack, as shown in Zone 5 (Figure 13f).

#### 3.4.3. Fatigue Failure Mechanisms of SPFC-AA Joints

**Figure 14:**
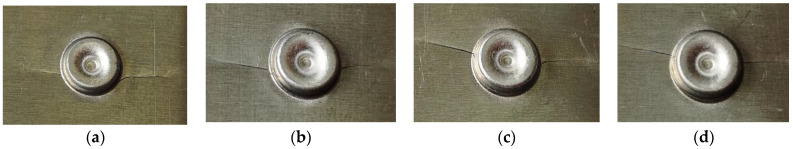
Failure forms of SPFC-AA joints with rivet heights of: (**a**) 6 mm; (**b**) 7 mm; (**c**) 8 mm; and (**d**) 9 mm.

The fatigue failure surface morphologies of SPFC-AA samples are shown in Figure 14. It can be seen from Figure 14 that no matter how high the rivet is, the fatigue failure occurs in the aluminum alloy, and the cracks are located near the joint button. The fatigue cracks appear on both sides of the joint button when the AA5052 is used as the lower sheet, as shown in Figure 14. The direction of the crack is approximately perpendicular to the direction of the fatigue load. It can also be seen that the cracks on the two sides of the rivets are almost at the center lines of the joint buttons. Due to the SPR, the effective cross-section of the AA5052 sheet is the smallest at the location of the cracks. The smaller the cross-section area, the greater the tensile stress, and the greater the tendency to crack.

**Figure 15:**
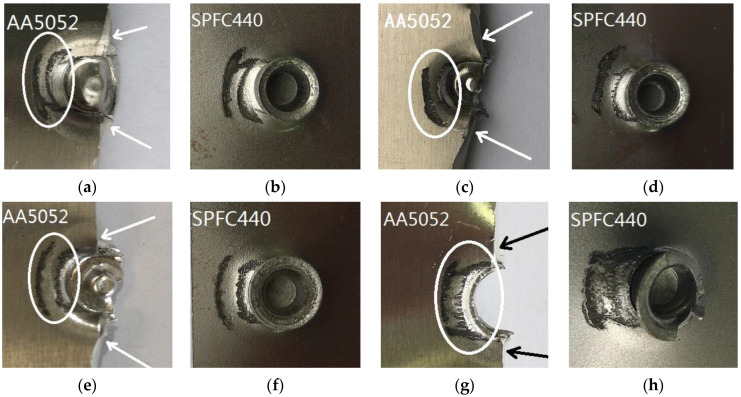
Wear morphologies of SPFC-AA joints: (**a**) AA5052 with 6 mm rivet; (**b**) SPFC with 6 mm rivet; (**c**) AA5052 with 7 mm rivet; (**d**) SPFC with 7 mm rivet; (**e**) AA5052 with 8 mm rivet; (**f**) SPFC with 8 mm rivet; (**g**) AA5052 with 9 mm rivet; (**h**) SPFC with 9 mm rivet.

Figure 15 shows the fretting of SPFC-AA samples under fatigue load. There are also obvious wear marks on aluminum sheets (marked by the circles in Figure 15a,c,e,g) and steel sheets (Figure 15b,d,f,h) near the rivet. The fatigue failure occurs on the aluminum alloy sheet, and the fatigue cracks are located on both sides of the rivet, as marked by the arrows in Figure 15a,c,e,g. The position of the crack sources is not consistent with the fretting position marked by the circle points. As can be seen from Figure 15, the friction degree of SPR joints with the rivet heights of 6 mm (Figure 15a,b) and 7 mm (Figure 15c,d) is more serious than that of 8 mm (Figure 15e,f) and 9 mm (Figure 15g,h). The reason is that the fatigue life of joints with rivet heights of 6 mm and 7 mm is longer (Figure 9), so the damage is more serious.

**Figure 16:**
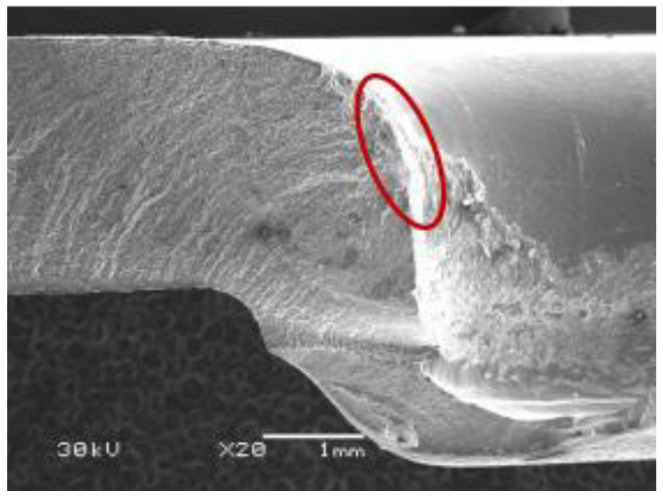
Cross-sectional fatigue crack of AA5052 alloy in SPFC-AA joint.

The fatigue crack of the fractured AA5052 is shown in Figure 16. The fatigue crack occurs not only between the upper and lower sheets, as pointed out by the arrow in Figure 4, but also between the lower sheet and rivet, as pointed out by the circle in Figure 4. The fatigue fracture of the lower sheet for SPFC-AA joints is located at the position marked by the circle in Figure 4. The fatigue crack originates from the fretting area of the contact surface of the lower sheet and rivet. At the same time, the lower sheet bends downward and stretches due to the rivet penetration force, and larger tensile stress is formed. Therefore, fatigue failure is related to tensile stress and fretting.

## 4. Conclusions

The joining properties of SPFC440/AA5052 multi-material SPR joints are investigated in this work. The influence of the rivet/sheet heights and the placing order of sheets on the SPR properties is emphasized. The main conclusions are following:

1. AA5052 aluminum alloy and SPFC440 high-strength steel can be joined effectively by the SPR process. The rivet leg flares symmetrically without cracks or buckling when the rivet height is 2–3 mm higher than the total thickness of metal sheets. There is an obvious crack in the rivet cross-section when the rivet height is more than 4.5 mm higher than the two layers of sheets’ thickness.
2. The rivet height and the laying order of metal sheets have some influence on the static tensile property of the SPR joints. When the aluminum alloy is used as the lower sheet and the rivet height is 8 mm, the SPR samples have the best static tensile property. The rivet legs fall off from the lower sheet in the tensile tests.
3. The rivet heights and placing order of materials affect the fatigue property of the SPR joint. The fatigue properties are excellent when the rivet is about 2.5 mm higher than the sheets. SPR joints have better fatigue properties when the harder steel material is used as the lower sheet. The failure mode is that the fatigue crack appears in the aluminum alloy. The initiation of the crack is related to fretting wear and tensile stress.

## Figures and Tables

**Table 1:** Chemical composition of AA5052 alloy and SPFC 440 steel (wt.%).

AA5052	Mg	Cr	Fe	Cu	Zn	Mn	Si	Al
	2.40	0.28	0.33	0.05	0.05	0.09	0.08	Bal.
SPFC440	C	Si	Mn	P	S	Al	Fe	
	0.24	0.28	0.33	0.05	0.05	0.09	Bal.	

**Table 2:** Mechanical properties of AA5052 and SPFC440.

	Tensile Strength/MPa	Conditional Yield Strength σ_0.2_/MPa	Elongation/%
AA5052	200	90	13.5
SPFC440	440	305	33

**Table 3:** Number of fatigue cycles.

Rivet Height	Laying Orders of Sheets	Fatigue Cycles (×10^3^)						
		No.1	No.2	No.3	No.4	No.5	No.6	Average Value
6 mm	SPFC-AA	108.8	104.7	88.5	76.0	80.8	99.1	93.0
7 mm	SPFC-AA	125.1	114.4	87.1	114.5	120.1	106.8	111.3
8 mm	SPFC-AA	92.2	71.4	61.6	68.6	62.2	67.7	70.6
9 mm	SPFC-AA	53.8	41.9	63.0	56.6	56.1	67.2	56.4
6 mm	AA-SPFC	204.5	122	108.7	117.1	115.1	133.8	133.5
7 mm	AA-SPFC	271.6	153.7	261.2	152.9	156.1	262.3	209.6
8 mm	AA-SPFC	129.7	107.5	173	170.3	162.6	142.4	147.6
9 mm	AA-SPFC	47.2	32.8	27.6	74.6	36.9	51.8	45.2

## Data Availability

The raw/processed data required to reproduce these findings cannot be shared at this time as the data also forms part of an ongoing study.
